# Supplementary material for: Beneficial Interactive Effects Provided by an Arbuscular Mycorrhizal Fungi and Yeast on the Growth of Oenothera picensis Established on Cu Mine Tailings
Source: Plants (Basel). 2023 Nov 29;12(23):4012. doi: 10.3390/plants12234012 (PMC10708390; doi:10.3390/plants12234012)
Supplement: Supplementary file 1 [file plants-12-04012-s001.zip › plants-2695358-SI.pdf]

Supplementary Table S1. Probability (p) values for the main effects and interaction for the variables measured and analyzed by means of a two-way ANOVA.

Variables	AMF	Yeast	AMFxYeast
Shot dry weight	.6186	.0056	.0001
Root dry weight	.0110	.0022	.0081
Chlorophyll A	.0000	.0000	.0000
Chlorophyll B	.0000	.0000	.0000
Carotenoids	.0000	.0000	.0000
Total Cu	.7054	.0002	.4855
DTPA-extractable Cu	.0000	.0000	.0347
Shoot Cu concentration	.3051	.0050	.6465
Root Cu concentration	.0445	.4321	.1652
Translocation factor	.0156	.2588	.0500
Superoxide dismutase	.5912	.0441	.0189
Catalase	.5675	.0026	.1035
Ascorbate peroxidase	.1524	.1813	.0116
Shoot total phenols	.2525	.0437	.1561
Shoot DPPH	.0698	.0000	.0000
Shoot Cuprac	.1436	.0188	.2536
Shoot ABTS	.0019	.1515	.0000
Root total phenols	.3661	.0011	.0001
Root DPPH	.9972	.0279	.0307
Root Cuprac	.7106	.0026	.0668
Root ABTS	.8130	.0089	.0152
